# Salutary Response to Targeted Therapy in Anaplastic Thyroid Cancer

**DOI:** 10.1177/2324709619890942

**Published:** 2019-11-26

**Authors:** Sasan Fazeli, Edina Paal, Jessica H. Maxwell, Kenneth D. Burman, Eric S. Nylen, Shikha G. Khosla

**Affiliations:** 1 George Washington University Medical Faculty Associates, Washington, DC, USA; 2 Washington DC VA Medical Center, Washington, DC, USA; 3 George Washington University, Washington, DC, USA; 4 MedStar Georgetown University Medical Center, Washington, DC, USA; 5 MedStar Washington Hospital Center, Washington, DC, USA

**Keywords:** anaplastic thyroid cancer, BRAF V600E, EZH2 mutation, CDK4 mutation, mitogen-activated extracellular protein kinase inhibitor (MEK inhibitor), BRAF inhibitor, targeted therapy

## Abstract

*Context*. Anaplastic thyroid cancer (ATC) is an aggressive tumor with a median survival of 3 to 9 months, a 1-year survival of less than 10% and without definitive therapies. Recently, in *BRAF V600E* mutated ATCs, new targeted therapy using a combination of a BRAF inhibitor, dabrafenib (Dab), with a mitogen-activated extracellular protein kinase (MEK) inhibitor, trametinib (Tram), has shown significant promise. *Case Description*. We report a case of aggressive ATC with 5 sequence mutations: *BRAF V600E* (mutation fraction [MF] 34%), *TERT E441del* (MF 37%), *RET N579K* (MF 55%), *EZH2 D154E* (MF 60%), and *CDK4 S259L* (MF 48%). The patient had a dramatic response to the Dab/Tram combination with near complete resolution of his lung, bone, hepatic, and splenic lesions soon after starting therapy. Unfortunately, intolerable side effects (grade 2-3) on this regimen required tapering and discontinuation of the treatment. He had a quick resurgence of disease after stopping the combination therapy. The patient died approximately 3 months after discontinuing Dab/Tram. Autopsy revealed an atrophic thyroid gland with microscopic subcapsular focus of well-differentiated papillary thyroid carcinoma. There was extensive lymphatic spread of the tumor throughout bilateral lungs with fibrosis. No other metastatic site was identified. *Conclusion*. We report a unique case of ATC with 2 new mutations of *EZH2 D154E* and *CDK S529L*. This case exemplifies the significant promise Dab/Tram therapy holds, the potential side effects that limit their use, and autopsy findings status post use of this combination therapy.

## Introduction

Anaplastic thyroid cancer (ATC) is a rare and aggressive malignancy that accounts for approximately 1.3% to 9.8% of all thyroid cancers globally.^1^ Regional and distant metastases are seen in 90% of cases at the time of diagnosis.^2^ Response rates to traditional chemotherapy, with or without radiation therapy and/or debulking surgery, are abysmal. ATC continues to be a lethal disease with previously reported median survival of 3 to 9 months and a 1-year survival rate of less than 10%.^3^

Recent advances in genomics have improved our understanding of this disease. Studies show that mutations that activate oncogenes and silence tumor suppressor genes contribute to its pathogenesis. Mouse models with *BRAF V600E* and *p53* mutations develop poorly differentiated thyroid cancers.^4^ In humans, *BRAF V600E* mutation is commonly seen in 20% to 50% of patients with ATC.^5^ Targeted combination therapy using a BRAF inhibitor, dabrafenib (Dab), with a mitogen-activated extracellular protein kinase (MEK) inhibitor, trametinib (Tram), has shown significant promise^6^ and has recently received an accelerated Food and Drug Administration approval.

## Case Report

A 69-year-old healthy, athletic male presented to ENT and Neurology with right-sided otalgia and headache (ECOG [Eastern Cooperative Oncology Group] grade 0). Over the next month, his symptoms progressed to neck pain, dysphagia, and a new rapidly enlarging right anterior neck mass. Thyroid ultrasound showed a 3.5-cm mass possibly arising from the right thyroid lobe. Computed tomography (CT) scan showed a soft tissue mass along the right aspect of the strap muscle, subcutaneous soft tissues with infiltration of skin. The mass was in contact with the right lobe of the thyroid gland but was thought to be anatomically separated. It did not arise in the right sternocleidomastoid muscle or in the sternoclavicular joint.

**Figure 1.**
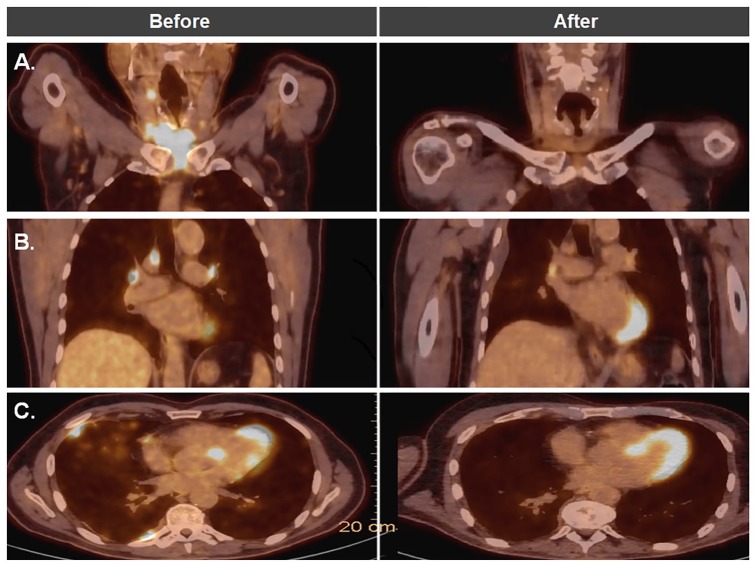
18F-FDG PET before surgery (before) and 4 months after initiating therapy (after): (A) Complete resolution of abnormal neck FDG uptake; (B) Complete resolution of 2 of 3 hilar lymph nodes uptake; and (C) Complete resolution of lung uptake.

An 18F-FDG-positron emission topography (PET) scan showed intense uptake in the neck mass extending to the surrounding musculature (SUV_max_ [maximum standardized uptake value] = 17.5), multiple neck lymph nodes (SUV_max_ = 10.7), right lung opacification (SUV_max_ = 8.4), mediastinal and hilar lymph nodes (SUV_max_ = 11.9), multiple muscle groups (SUV_max_ = 15.0), and right posterior 11th rib (SUV_max_ = 6.5; Figure 1).

Fine needle aspiration biopsy revealed large cohesive sheets of overlapping highly atypical epithelial cells, signifying poorly differentiated carcinoma of uncertain origin. Subsequent skin mapping punch biopsy showed a poorly differentiated carcinoma of uncertain origin infiltrating skeletal muscle, dermis, and subcutis. Subsequently, immunostains of both fine needle aspiration and punch biopsy samples showed tumor cells positive for CK7, AE1/AE3, and focally positive for CK5/6 and negative for CK20, thyroid transcription factor-1, thyroglobulin, CEA, chromogranin, synaptophysin, CD56, CDX2, RCC, napsin-A, S-100, and p16. The patient soon developed an ulcer over the neck mass. Punch biopsy of this ulcer showed tumor cells with the same morphologic characteristics and Ki-67 20% positive. The working diagnosis at the time was primary adnexal carcinoma versus metastatic carcinoma from lung, pancreas, liver, kidney, or adrenal gland.

The patient underwent tumor debulking with a myocutaneous advancement flap for local control and symptomatic relief. The involved skin and underlying tumor were resected. There was a fascial plane between the tumor and the thyroid gland; therefore, the tumor was resected off the thyroid gland and trachea. Frozen section margins confirmed that tumor invaded into internal jugular vein and the trachea. Final pathology revealed ATC with metastatic carcinoma in 7 of 15 resected lymph nodes. Few foci in the neck mass and more obviously in the lymph node metastases showed well-differentiated papillary thyroid carcinoma (PTC). The presence of the well-differentiated foci aided the establishment of the final diagnosis. Immunostaining was positive for thyroid transcription factor-1 and thyroglobulin in the well-differentiated tumor component; with focal positivity in the transition zone of the tumor and no reactivity in the area with the anaplastic carcinoma. The anaplastic tumor retained PAX-8 reactivity. Based on these findings, the punch biopsy sample was consistent with PTC.^7-9^

He was diagnosed with metastatic ATC stage IV-C as per the American Thyroid Association 2012 guidelines.^10^ Genetic analysis with 125 gene panel (Personal Genome Diagnostics, Baltimore, MD; http://www.personalgenome.com/) for microsatellite instability, sequence mutation, amplification, and translocation revealed 5 sequence mutations: *BRAF V600E* (mutation fraction [MF] 34%), *TERT E441del* (in-frame deletion; MF 37%), *RET N579K* (MF 55%), *CDK S259L* (MF 48%), and *EZH2 D154E* (MF 60%). Programmed death ligand receptor-1 immunochemistry was positive in 20% of tumor cells (Quest Diagnostics, Chantilly, VA).

Postsurgery, the patient suffered wound dehiscence twice. He became home oxygen dependent (ECOG Performance Status, grade 3) and his CT chest showed progression of the disease with bilateral pleural effusions. Given his rapid deterioration, distant metastases, and positive *BRAF V600E* mutation, he was started on combination of oral Dab 150 mg twice daily, Tram 2 mg daily (Dab/Tram), and dexamethasone 8 mg daily on compassionate grounds.^6^

The patient had a dramatic response to Dab/Tram combination treatment. At week 8 of Dab/Tram therapy, he had resolution of pleural effusions and normalization of oxygen saturation at room air with normal performance status (ECOG grade 0). CT scan showed significant reduction of several lung nodules, near total resolution of bilateral basal lung consolidations, reduction in size of multiple mediastinal, hepatic, and splenic lesions. This was consistent with near complete response (partial response by RECIST criteria 1.1). Soon after initiation of therapy, the patient developed high-grade fever of 105°F for about 30 minutes after taking Dab (Grade 3, by Common Terminology Criteria for Adverse Events Version 5.0 [CTCAE]). This was controlled with acetaminophen prophylaxis. He also had an episode of painful (10/10 in intensity) oral mucositis (Grade 3 CTCAE), which was treated with magic mouth wash. The mucositis resolved after holding the medication for 3 days and therapy was resumed. The valacyclovir prophylaxis was initiated.

The follow-up PET/CT scan, 16 weeks after initiating therapy, showed complete resolution of abnormal neck, lung, and 11th rib uptakes as well as majority of musculature uptake. The hilar lymph node uptake decreased to SUV_max_ of 5.3, and right thigh SUV_max_ decreased to 3.2 (Figure 1).

At week 27 of initiating Dab/Tram therapy, the patient was pain free, active, and gaining weight. A week later, the patient was admitted with continuous high-grade fever of 105°F (Grade 4 CTCAE), elevation of liver enzymes to approximately twice the upper limit of normal (Grade 1 CTCAE), and a lowering of platelet count to 99 000/mm^3^ (Grade 1 CTCAE). Infectious etiology was ruled out. Dab was held and patient’s pyrexia resolved. The patient subsequently developed an erythematous acneiform papulopustular rash with areas of confluent plaques on his head, neck, and upper torso (Grade 2 CTCAE). At week 29, Dab was restarted at a lower dose of 150 mg once daily. By week 33, while on the lower dose of Dab, the patient had recurrence of high-grade fevers (Grade 3 CTCAE), progression of his rash (Grade 3 CTCAE), and developed floaters in his right eye (Grade 1 CTCAE). A week later, both Dab/Tram were stopped by the patient, and he wanted to consider other treatment options. Repeat PET scan at week 37 showed new left upper lung lesions with hilar lymphadenopathy. At week 38, he underwent an endobronchial ultrasound-guided bronchoscopy for his hilar lung lesion that showed metastatic disease, and a week later, he developed a postobstructive pneumonia. At week 40, he was started on cabozantinib 60 mg per day; however, 4 weeks later, he had progression of his lung nodules, pleural effusion, significant calf and back pain. The pain was likely from metastatic spread of tumor to the spine and possibly as a side effect of cabozantinib. The patient was admitted to the hospital at week 46 with extensive tumor spread and pulmonary emboli. He died of respiratory failure 48 weeks after initiating Dab/Tram therapy, 13 weeks after discontinuing Dab/Tram and 8 weeks after starting cabozantinib.

His most significant autopsy finding was extensive lymphatic spread of ATC throughout his lungs bilaterally with infiltration of adjacent connective tissue without formation of tumor nodules, associated with dense focal acute inflammatory infiltrates and areas of necrosis. A tumor embolus was noted in a small branch of the pulmonary artery that was associated with infarction of the adjacent lung parenchyma. There was also significant fibrosis throughout the lungs. The thyroid was atrophic and was difficult to explore due to scarring of the surgical site. The entire thyroid gland was examined microscopically. Histology showed presence of a microscopic focus of well-differentiated PTC in the subcapsular region of the right lobe with intraglandular spread, as well as infiltration of the tumor through the capsule into the surrounding connective tissue. Several lymph nodes showed metastatic well-differentiated PTC.

## Discussion

The current case illustrates the profound impact mutation-targeted therapy has on the clinical course of ATC. Recent advances in our understanding of thyroid cancer, gene pathways have been vital to utilizing these therapies in its treatment.

Our patient had a rapid and significant reduction in tumor burden while he was able to tolerate the combination Dab/Tram therapy. The rapid tumoral response was not without notable clinical challenges. The patient developed an erythematous acneiform maculopapular rash with a seborrheic distribution most likely due to this drug combination as it improved with a drug holiday. The patient also experienced significant symptomatic pyrexia with fevers to 105°F, interrupting his treatment. Unfortunately, within 1 month of discontinuing the treatment, he showed significant resurgence of the disease. His disease did not respond to cabozantinib monotherapy. He expired 13 weeks after stopping Dab/Tram combination therapy. This indicates that perhaps there is a small window of opportunity to starting Dab/Tram therapy in patients with this rapidly progressive lethal disease without interruption. Further studies need to be done for optimum management of drug intolerability and indications for drug holiday to allow use of this combination therapy to its full potential.

There is paucity of literature on the autopsy findings in ATC patients on combination therapy. In our patient, the well-differentiated PTC was seen in the thyroid tissue but only a small amount of ATC was present. This was perhaps due to higher response rate of undifferentiated tumor cells to this combination.

About 20% to 50% of ATC harbor activating *BRAF V600* mutations.^6^ It has been demonstrated in mouse models that combined inhibition of BRAF and MEK kinase enhances antitumor activity compared with single-agent BRAF inhibitors in *BRAF V600* mutant mice.^4^ This combination therapy has been successful in treating *BRAF V600* mutant melanoma and lung cancer.^11,12^ Importantly, Subbiah et al, in a phase II, open-label trial, on a cohort of 16 patients with predefined *BRAF V600E*-mutated ATC, showed the BRAF inhibitor Dab and MEK inhibitor Tram improved the overall response in 11 of 16 cases (69% response rate, 95% confidence interval = 41% to 89%), with median follow-up of 47 weeks, and 7 ongoing responses.^6^

Enhancer of Zeste Homolog 2 (EZH2) has only recently been described in ATC and appears to play an important role in tumor growth.^13^ Borbone et al showed that EZH2 is specifically overexpressed in ATC cell lines, and directly controls differentiation of ATC cells by silencing the thyroid-specific transcription factor paired-box gene 8.^13^ They also demonstrated that knockdown of EZH2 in ATC cell lines results in cell growth inhibition, loss of anchorage-independent growth, migration, and invasion properties.^13^ Cyclin-dependent kinase 4 (CDK4) upregulation has been noted in aggressive thyroid cancers and their role in mitotic activity of such thyroid tumors is of great interest.^14^ CDK4 protein plays a key role in the cell cycle progression across the G1/S phase transition.^15^ A preclinical study of CDK4/6 inhibitor, ribociclib in PTC, and ATC cell lines has been shown to inhibit tumor growth with decreased expressions of pRB, pAKT, and Ki-67, and significantly increased tumor cell apoptosis. In our literature search on Medline and PubMed, we did not find any cases of ATC with *EZH2 D154E* and *CDK S259L* oncogenic mutations. In the Catalogue of Somatic Mutations in Cancer database (COSMIC v89, 2019), none of the 409 ATC samples that were tested for EZH2 or the 264 samples tested for CDK4 harbored these mutations (https://cancer.sanger.ac.uk/cosmic/). In the National Institutes of Health ClinVar database for Genomic variations (https://www.ncbi.nlm.nih.gov/clinvar/) EZH2 *D154E* has not been described and the *CDK4 S259L* has not been seen in any thyroid cancers. The functional consequences of missense mutation EZH2 D154E, and the CDK4 S259L mutation, that were seen in the current case, are unknown and there is no approved targeted therapy for these mutations.

In summary, the combination of proto-oncogene BRAF inhibitor Dab and MEK inhibitor Tram offers promise in the *BRAF V600E*-mutated ATC patients. The Dab/Tram combination displayed a potent and rapid clinical response in our patient although new challenges in his management are evident. This degree of salutary response rate has not been previously reported in ATC. This 2-drug combination is now Food and Drug Administration approved for treatment of *BRAF V600E* mutation-positive ATC. Studies using Dab/Tram therapy with additional check point inhibitors in a 3-drug cocktail are currently underway. Further research efforts are needed to better manage the side effect profile of this combination therapy and to understand the role the EZH2 and CDK4 mutations play in the clinical progression and management of this disease.
